# The Pendulum Has Swung: How Do We Ensure a Life Course Approach to Immunisation in Australia?

**DOI:** 10.3389/fpubh.2021.801176

**Published:** 2022-02-04

**Authors:** Holly Seale, Kathleen McFadden, Amalie Dyda, Jessica Kaufman, Anita Heywood

**Affiliations:** ^1^School of Population Health, Faculty of Medicine and Health, University of New South Wales, Sydney, NSW, Australia; ^2^Centre for Applied Health Economics, Griffith University, Southport, QLD, Australia; ^3^School of Public Health, Faculty of Medicine, The University of Queensland, Herston, QLD, Australia; ^4^Vaccine Uptake Group, Murdoch Children's Research Institute, Melbourne, VIC, Australia; ^5^Royal Children's Hospital, Melbourne, VIC, Australia; ^6^Faculty of Medicine, Dentistry and Health Sciences, University of Melbourne, Melbourne, VIC, Australia

**Keywords:** immunisation, vaccine, acceptance, attitudes, communication

## Abstract

Rather than concentrating primarily on children and adolescents, there has been a shift in the discourse around immunisation to encompass a whole-of-life approach. Despite this acknowledgement and ongoing high burdens of vaccine preventable diseases in adults, coverage for some adult risk groups remains sub-optimal. This study aimed to explore key informant's and stakeholder's perceptions of factors impacting provision of immunisation programs for Australian adults and to identify strategies to promote acceptance and uptake. Semi-structured telephone interviews were undertaken with people involved in adult immunisation program delivery, advocacy, policy or research between September 2020 and June 2021. Transcripts were inductively analysed, with the resulting themes categorised into the five influences on vaccination gaps that have informed program planning in other countries: Access, Affordability, Awareness, Acceptance and Activation. Participants spoke of improvements in the provision of vaccines to adults, however, ongoing challenges persisted. Participants agreed that the focus or emphasis of policies and the promotion/communication strategies has been on childhood vaccination in Australia, however there is a sense that the “pendulum has swung.” These included understanding of eligibility amongst the Australian population and the reluctance of some health providers to dedicate time to exploring immunisation needs with adult patients. In comparison to the childhood vaccination program, there has been a lack of data available on coverage for adult vaccines on the national immunisation program. This has contributed to the ongoing challenges of identifying and promoting certain vaccines. At a government level, questions were raised about why the Australian government has never set an aspirational target for adult vaccination (i.e., influenza or pneumococcal) coverage. While significant improvements have been made in adult immunisation uptake, there are still gaps across the program. While the system remains under stress because of the COVID-19 pandemic, it is not appropriate to implement any additional programs. There needs to be strong commitment to establish the value of adult vaccination in the eyes of community members, policy makers and healthcare professionals. Having a national adult immunisation strategic plan would help advance action.

## Introduction

Complete and timely data has only just started to become available on adult vaccination as an outcome of the expansion of the Australian Immunisation Register (AIR). Prior to 2016, the AIR (formally the Australian Childhood Immunisation Register) only recorded childhood vaccines up to 7 years (1). Shifting to a whole-of-life register was done to capture adult vaccinations including maternal influenza and pertussis vaccines for pregnant women and vaccines for medically/aged-related risk groups. It should be noted that the AIR coverage data is unable to capture vaccine coverage for adults with medical conditions, it can only provide data by age eligibility. While an early analysis has revealed gaps in the completeness of the data, it has also confirmed the findings from smaller studies that coverage of recommended vaccines for adults needs improving (2, 3). As an example, while influenza vaccination coverage estimates for adults aged ≥ 65 years have been 70% or greater, coverage for adults with certain medical conditions, such as severe asthma, lung or heart disease and diabetes is as low as 45% (4). This is despite influenza vaccination being free for these target groups and improving vaccination coverage in high-risk groups being a national strategic priority. Approximately 23 months following commencement of the national zoster immunisation program, cumulative vaccine uptake was 31.2% for adults aged 70–79 years old, with a slightly higher vaccination uptake documented for Indigenous adults (37%) (5). This number is likely an underestimation of the true zoster vaccine uptake, as another study (based on primary care data) has documented the vaccine coverage as being between 41and 46% (6). Despite this variation in the numbers, both reports suggest that uptake is suboptimal. Lastly, pneumococcal vaccine is recommended for non-Indigenous adults aged ≥70 years, Indigenous adults ≥ 50 years and for people with risk conditions from >12 months of age. A systematic review of Australian studies from 1992 to 2013 estimated that pneumococcal vaccination coverage for people ≥65 years ranged from 50.3 to 72.8% after the introduction of the vaccine onto the national program (7).

The Australian Government recommends and funds several vaccines for adults as part of the National Immunisation Program including against shingles for all adults aged 70–79 years or more, pneumococcal for those aged 70 years and over, as well as influenza for people 6 months and over with specified medical risk conditions, pregnant women (as well as pertussis) and all people over 65 years. For Indigenous adults, the pneumococcal is funded for adults from 50 years and over. In addition, all people aged < 20 years are eligible for free catch-up vaccines (8). The COVID-19 pandemic has brought a renewed focus on not only adult immunisation, but on equity in immunisation provision. One suggested strategy to ensure all target groups receive relevant vaccines is to identify additional settings and providers to promote, recommend and deliver immunisations. Our earlier work indicates the potential for hospital-based interventions for improving opportunistic inpatient vaccinations (9). However, currently, hospitals are currently be underutilised in Australian for adult vaccination, despite the willingness of the public to receive vaccinations in this setting (10). Another setting that is current underutilised is community pharmacies as immunisation providers, accounting for only 2.9% of vaccine provision in 2019 (11). New legislation now allows pharmacists to deliver a wider range of vaccines in some Australian States and Territories (5). Given the expanded network of immunisation providers used to deliver the COVID-19 vaccine program in Australia, is it time to focus efforts on technology and other support systems to support adult vaccination efforts in settings other than primary care. The aim of this study was to explore key informant's and stakeholder's perceptions of factors impacting provision of immunisation programs for Australian adults and to identify strategies to enhance opportunities to improve coverage.

## Methods

Semi-structured telephone interviews were undertaken with people involved in adult immunisation program delivery, advocacy, policy, or research in Australia. Interviews were approximately 30–40 min in duration, between September 2020 and June 2021. The Human Research Ethics Advisory Panel (social/medical) at the University of New South Wales reviewed and approved this study (HC190799).

### Sampling

Key informants were defined as those people who have an active role in the delivery of adult immunisation programs across settings (including GPs, practise nurses and pharmacists), while stakeholders were people involved in immunisation-related advocacy, policy/program development or research.

Participants were recruited to the study via two approaches. Firstly, an online search of relevant websites was conducted to identify potential candidates matching the selection description. Each candidate was then followed up via email with an invitation letter. Secondly, interested candidates were asked to directly recommend any colleagues who would be willing to participate as well. Participants were only enrolled into the study when full verbal consent had been received. An effort was made to recruit at least one participant from each of Australia's States and Territories to capture a broad range of views encompassing State-based differences in policy. This study did not collect any identifiable personal information from the participants.

### Data Collection

An interview guide was jointly developed and reviewed by the researchers to identify key areas of interest for the study (Table 1). Given the timing of the study, the interview guide was amended to include questions about COVID-19 vaccine delivery and communication. The interview guide included a series of questions related to the following topics: factors impacting on vaccine uptake and delivery for Australian adults, perceptions toward current efforts to promote and deliver vaccines to adults, future strategies that could be used to improve awareness and uptake, attitudes toward alternative settings for vaccine delivery, and barriers and facilitators impacting in- or out-patient immunisation delivery. The list of topics served only as a general direction during each interview. In addition, the interviewer used paraphrasing and additional questions to seek clarification. This ensured that the study included most of the topics and was flexible to changes depending on the actual scenario. Questions were asked in an open-ended manner to allow room for expansion. For example, interviews often began with a broad question like “what are your thoughts about the use of the influenza vaccine in hospitals?” to allow participants to freely discuss their opinions. Prompts were only given when the interviewer deemed, they were required to encourage the conversation back to topic. During the interviews, member checking was conducted to ensure that the ideas identified during the early phase of analysis were appropriate. We identified emerging themes through analysis of the first 10 interviews, then continued to sample, following identified leads until we reached thematic saturation. This meant we reached a point where no additional issues or insights emerged from the data and this redundancy signals that data collection may cease (12).

**Table 1:** Interview guide.

How do you feel the Australian community perceives the need for adult immunisation
What factors do you think impact on delivering immunisation programs to Australian adults?
What do you think of the current efforts around the delivery/promotion of vaccination to Australian adults?
What strategies/interventions do you think could be used to promote immunisation to Australian adults?
Who do you believe should play a role in the promotion and delivery of adult vaccination to the target groups?
What are your thoughts on promoting/delivering vaccination in other settings?

### Data Analysis

The interviews were recorded, transcribed verbatim and inputted into QSR NVivo® Version 12 to facilitate analysis. HS (prior research on influenza vaccination) was responsible for data analysis. The interviews were repeatedly listened to, and notes were taken to ensure that all the relevant ideas had been captured. An iterative process of data analysis was conducted. The initial phase involved familiarisation via multiple readings of the interview transcripts. The transcripts were coded initially, and a coding scheme developed. To ensure a true representation of the participants opinions and experiences, we attempted to use the participants voice in describing in the naming and description of the codes. Each of the codes were then attributed to the five influences (5A's) on vaccination gaps that have informed program planning in other countries: Access, Affordability, Awareness, Acceptance and Activation (13). Compared to other classifications of determinants of uptake, the authors aimed to ensure that the taxonomy was simple and instinctive, with the express aim of supporting the development of a mutual understanding of a complex problem. We followed the consolidated criteria for reporting qualitative research (COREQ), which consists of 32 items grouped into three domains: (i) research team and reflexivity, (ii) study design and (iii) data analysis and reporting (14).

## Results

A total of 17 interviews were undertaken with stakeholders from across Australia. This included personnel such as immunisation researchers (*n* = 5), those in non-government advocacy roles (*n* = 2), people working in local health departments/public health networks (*n* = 3) and in the primary- or tertiary care settings (*n* = 7).

### Awareness and Acceptance

There was a level of consensus amongst participants that the focus or emphasis to date has been on childhood vaccination in Australia, or on adults aged 65 years and above (“*tiny tots and older adults*”). As outlined by one participant, the government took the view that it was “*hard to get people in high school, once you moved beyond high school, it was too hard*” (*Participant 10, Healthcare provider*). For people between the age of 18 to 50 years there are far less opportunities to vaccinate. Across most of the interviews, participants spoke about a lack of coverage data as one major factor limiting awareness of and engagement with adult vaccination. They did acknowledge that in the future data should be more available due to changes in the Australian Immunisation Register and the requirements for vaccines on the national program to be recorded.

“*It definitely gets considerably less discussion and weighting compared to childhood immunisation and I think a lot of that may be since we haven't had very good data on it. We've had childhood immunisation coverage rates for 20 years and quarterly reports from the Commonwealth and annual reports and we do that and there's a bit of a hole…. before you even get to start talking about how you can improve uptake, you got to be able to measure it accurately so that you can then report that back to the public and the providers and get a bit more of that feedback loop because at the moment there is a little bit of a vacuum in some ways” (Participant 11, Immunisation researcher)*.

But there was a consensus amongst the participants, that the “*pendulum has swung*.” The introduction of the HPV vaccine raised awareness of adolescent vaccination. Beyond that the Zostavax and the pneumococcal changes were suggested to be one of the drivers to this change, while the focus on vaccination during pregnancy may have also contributed. Lastly, COVID-19 was acknowledged as an opportunity to think “*more broadly to improve coverage*.”

However, of concern was the suggestion that there are still issues with adult vaccine acceptance and negative attitudes towards adult vaccination: “*there are some general practitioners who do not believe in the efficacy of vaccines (for adults), or at least don't believe they're as efficacious as they need to be*.” It was suggested that we need greater awareness about vaccination “*amongst specialist groups, not just geriatricians but general physicians, respiratory physicians*” *(Participant 7, Healthcare provider)*.

At a population level, the suggestion was that most people support “*the notion of vaccination*,” but people are not aware of their eligibility for a vaccine or are agnostic about the need. Participants who were responsible for delivering immunisation programs in high migrant areas strongly endorsed the sentiment that migrants are “*very pro-vaccine*,” usually as a result of living in countries “*where these diseases are rampant*” or because they know someone who has had the disease. However, there are others in the community who do not see themselves as being in a risk group despite having certain health conditions: “*…we know that certain risk groups qualify for a free vaccine, but there are a large number of people that do not see themselves as being in that risk group despite having certain conditions” (Participant 6, Non-government organisation)*.

A subgroup of adults that were identified as needing additional resources and support were those who have previously self-identified as having an anaphylaxis to a past vaccine and stating that they are not eligible for future vaccines. These adults may become hesitant to receive a vaccine in the future because of these past events. It was also suggested that more needs to be done to support those with needle phobias.

“*You've got an unusual health condition, a particular health concern, talk to your GP, but your GP might not feel comfortable giving that vaccine. Having that referral service for that sort of support, and typically that would be through the State or Territory Health department to talk about that, and then it will be kind of a network thing of who they know who are able to do that kind of work. I think that a concept of that sort of specialist vaccination clinic, where that's needed, is one issue” (Participant 10, Healthcare provider)*.

### Access

Participants spoke of improvements in the provision of vaccines to adults. With the delivery of vaccination by nurse practitioners, pharmacists, hospital-based doctors, and Aboriginal Health Workers, in addition to GPs, it should be theoretically easier to get vaccinated. However, it was acknowledged that there is still strong resistance amongst GPs to pharmacy-based vaccination and that there is “*fundamental misunderstanding by both groups about the other group*.” It was also suggested that international settings (examples given UK and Canada) are ahead of Australia in terms of delivery of vaccines via pharmacy-based settings. On participant said: “*Pharmacists are much more autonomous. The information systems are streets ahead. They have much better records than we do. They do have targets.”* Beyond these settings, workplace, and community-based pop-up clinics (i.e., Town Hall clinics) were also identified as important vaccination opportunities. Home service opportunities were also suggested to capture those people in the community living with disabilities, or older people or those who find it hard to access vaccination. None of the participants mentioned hospitals as a site unprompted but when questioned had mixed responses to the suggestion. Once prompted, some acknowledge that it may be a useful site to capture people “*incidentally*” but that it would be a limited subset of people and that it would be costly. There were other participants who felt very strongly about the need for hospital-based vaccination programs: “*1000%, hospitals should be vaccinating inpatients*.” One of the key issues raised was about the funding of the vaccine, as hospitals cannot charge Medicare while the patient is still classified as an inpatient. One solution that was proposed was to include vaccination as part of the patient discharge process.

“*I think Australia is just so far behind other countries regarding what vaccines can be offered in the pharmacy setting. They should be giving pneumococcal vaccines, particularly those vaccines that aren't funded for people like the diabetic. They see these patients probably more often than the GP does. I think it's really important that we here in Australia step up and get with the rest of the world program and just expand that scope of practise for pharmacists and give them access to these funded vaccines” (Participant 2, Government)*.

“*I would be pro-consumer choice, in terms of where it's available. I think it should be available in as many avenues as possible because we would want the roll out to be as quick as possible, and to be as accessible as possible. I think it's a very good opportunity to potentially trial things that haven't been trailed yet. Is there an at home service for those more vulnerable, people living with disabilities, older people, people who find it hard to access it?” (Participant 4, Researcher)*.

“*We do have to try and move the way some people think about the delivery of vaccines. I think we need to bring GPs and pharmacists to the table. We've done this before and we witnessed in those meetings that there is a fundamental misunderstanding by both groups about the other group. I think there's a lot of work that can be done in that space.” (Participant 6, Non-government organisation)*.

One of the challenges in trying to understand access concerns is linked to the lack of data. As outlined by one participant we cannot currently model the impact of changing the vaccine delivery system, as we do not have good denominators on who is vaccinated. This is a key puzzle piece that has been missing, but with the revisions to the AIR, there should finally be data to determine where the gaps are.

### Affordability

The cost of vaccination for an individual was not raised by any of the participants, beyond noting that some members of the community can have access issues in reaching immunisation settings.

“*Vaccines are a very equitable intervention, but access to vaccines is not always equitable. There are people who lack transport or who can't get to facilities or in different areas it's tougher to get some vaccine. You might go to a GP and the vaccine be free, but the consultation is not, and those are difficult spaces” (Participant 10, Healthcare provider)*.

### Activation

Amongst the participants, GPs were acknowledged as having an important role in advocating for vaccination but also for supporting people's decision-making. However, it was also conceded that not everyone goes to see their GP. On the flip side, it was suggested that not all GPs have the time to “*open pandora's box*” and delve into the vaccine history of adults: “*If the patient has come in for another reason, whether it's just a repeat prescription or they've got a mild illness or something like that, the providers are just going to look at that particular reason for presentation” (Participant 2, Government)*. As one participant suggested “*if the patient wants it, they will ask for it*” but also acknowledged that people don't want it, then some providers won't persevere on discussing the need for the vaccine. This may be due to the skill set of the provider or may be because of an issue of time.

“*I will always say to providers, ”If John Smith refuses your zoster vaccine this time, next time you see John Smith, you'll be asking him again, and you keep doing that. If you see John Smith nine times, you ask the same question nine times." But I don't believe that that really is happening. I think that they've ticked the box to say that they've offered it to all of their patients and that's the end”(Participant 2, Government)*.

Having an accurate immunisation history of vaccine uptake available to the GP may help down the track, as well as system updates to remind the GP. One suggestion was to expand the health cheques or care plans done to younger adult groups so that vaccination is formally checked. Lastly, incentives to the GP may also help to increase adult vaccination. Issues were also raised that there is a lack of visual resources promoting adult immunisation within clinics. It was acknowledged that promotional materials are produced by the vaccine companies for vaccines but that “rarely will you find more generic resources around the importance of vaccination.” There are even cases where general practises do not want to put posters on the walls. This means that “*If those providers aren't verbally recommending those vaccines, then realistically the public don't realise that they need them*” *(Participant 2, Government)*.

There is a need to work with local communities especially community leaders. Within the clinics, it was also proposed that dedicated days could be given to vaccinating certain culturally and linguistically diverse community members, so that translators or bilingual health professionals can be onsite to support communication. “*I think your biggest challenge is getting the community leaders to really educate their population. Getting them on side is really important” (Participant 3, Healthcare provider)*.

At a government level, questions were raised about why the Australian government has never set a population vaccination target for relevant vaccines. One participant felt that this resulted in “*mixed message both to practitioners and to consumers*.” Whereas other countries have been clearer about population level targets for adult vaccines. One reason given for the lack of targets was that there was not sufficient pressure to set one for adult programs, compared to the childhood program. Beyond the drive to set targets, concerns were raised about the consequences of not meeting them and whether it would spur GPs into action.

“*I think setting targets is a great idea. I think there should be targets that are set. It would then potentially prompt providers to have those conversations with adults around adult vaccination. If there are targets set up, who cares? What's the incentive or what– Who cares if I don't reach 90%? What happens? I'm not going to get deregistered, or my practise isn't going to close down. There's no consequences to not reaching the targets” (Participant 2, Government)*.

Comments were also made about the level of resources provided into developing resource and communication strategies. Lastly, participants also identified that there is more work needed regarding the level of promotion and advocacy done by peak bodies and support organisations about vaccination. One participant suggested that the peak bodies could have a “*stronger stance around vaccination. I think their messaging is either absent or very soft.”*

“*The [peak body] we know do promote vaccination, particularly influenza vaccination that we worked with quite recently…. There are some [peak bodies] that are a little bit sceptical. We need a way to engage with these groups and to bring them to the table so that we're all singing off the same hymn sheet” (Participant 6, Non-government organisation)*.

## Discussion

As outlined in the interviews, the absence of data for adult vaccine coverage continues to have an impact on the promotion of adult vaccination. Coverage data for childhood vaccination has been available (and GPs incentive to report to the system) since 1996 and so has helped to shape discussion and policy decision making. Whilst vaccine coverage data for adults (for vaccines given under the national immunisation program plus influenza and COVID-19) will become more reliable soon, an issue remains with the usefulness of the system for tracking coverage in at-risk groups. Currently, the AIR data does not capture a person's at-risk status including whether a person has a medical risk factor or are pregnant, and it is not feasible to track coverage by these important risk factors (1). Having the capacity to identify those with conditions that make them eligible for vaccination (especially those under 65 years), would also enable more targeted estimates of vaccine effectiveness for at-risk individuals, allowing a more comprehensive assessment of targeted programs.

With the availability of enhanced data, it also opens the opportunity to set benchmarks for the States/Territories to reach for adult vaccine coverage. Currently the National Partnership on Essential Vaccines (an agreement between the Commonwealth of Australia and the states and territories), currently includes three benchmarks related to vaccine coverage: (1) an increase in vaccination coverage rates for 60– <63 month olds; (2) an increase in the vaccination coverage rates for Aboriginal and Torres Strait Islander children; (3) an increase in vaccination coverage rates for 60– <63 month olds in low vaccine coverage geographic areas (15). Given the aim of the benchmark is to “protect the Australian public from the spread of vaccine preventable diseases through the cost-effective and efficient delivery of immunisation programs under the National Immunisation Program,” it would be within scope to expand this benchmark to include one vaccine directed at adults. One example would be to focus on ensuring equity in influenza vaccine coverage for people aged 65 years. Evidence shows that countries that have specific objectives defined for their vaccination programs also report higher levels of uptake (16). The National Adult and Influenza Immunisation Summit (NAIIS), a multi-stakeholder coalition of federal and non-federal partners working to increase adult immunisation coverage rates for recommended vaccines, has recently sought to develop new quality measures for adult immunisation. The working group that was formed for the summit argued that having “valid, actionable, auditable, and relevant benchmarks available for measurement is central to align incentives for adult immunizations” (17).

There was a consensus that having a broad range of immunisation providers (and convenient settings) was a positive step for the promotion of adult vaccination. Despite that, most of the focus still tended to focus on GPs when it came to talking about promoting and delivering vaccines to adults. Beyond those settings, there was very little discussion regarding the role of workplace, local council clinics, pop-up clinics, patient outreach (e.g., home visits or group visits to health professionals) and lastly hospitals as places to promote and deliver immunisation. When it comes to actual settings of vaccine delivery, only recently has there been attempts to try and map where adults are receiving their vaccines (outside of primary care). Trent et al. undertook one such study looking at influenza vaccines administered in non-medical settings and found that 13% received it at a pharmacy and 14% at their workplace (18). Perhaps not surprising that a greater proportion of workplace vaccination occurred in those under age 65 compared to those over 65 years, while adults without chronic health conditions were more likely to get vaccinated in non-medical settings compared to those with chronic conditions or aged 65 and over. Cost and convenience were the two main drivers of why people were vaccinated at their workplace. Similar concepts around convenience, ease of access, cost, bookings not being required, timeliness were the key reasons why people chose a pharmacy-based vaccine opportunity (18). The authors concluded by suggesting that non-medical settings such as workplaces and pharmacies “may be an enabler of vaccination by making it accessible to working people who may not feel they have time to see a doctor for vaccination” and that further work is needed around how best to promote vaccine uptake in non-medical settings.

Amongst the participants there were very mixed views regarding the value and role of hospitals. While some participants were overwhelmingly positive about the need to promote hospital-based vaccination more, others were very reluctant. Issues around the capacity to provide immunisation services including whether they have sufficient cold chain infrastructure and staff training through to the culture of the service and the focus on acute care. While hospitals may not be perceived as being suitable for the delivery of vaccination, the idea of promoting vaccines at the hospital was certainly embraced and that efforts should be made to ensure that vaccine recommendations get included on discharge summaries and correspondence occurs between hospital specialists and GPs to ensure follow up. This aligns with the recommendations made by Michel et al. that any contact with the healthcare system is an opportunity to discuss vaccination and to cheque status (16).

Regular scheduled child health cheques, vaccination record booklets and appointment reminder schemes have been used to nudge parents to ensure their children are vaccinated as per the schedule. However, these aspects of the immunisation program are not always replicated for adults. This issue was recently highlighted in a systematic review looking at the value and impact of nudges (entail changing vaccine defaults (optout), giving incentives, and providing reminders/recalls) to address vaccine hesitancy (19). The review included 48 published papers, of which only 14 focused on adults including pregnant women, college students and those aged ≥65 years. The authors concluded the paper by suggesting that nudge-based interventions show potential to increase vaccine confidence and uptake, however, further evidence is needed to develop clear recommendations.

One suggested opportunity was to explore the use of health plans as potential ways to nudge providers to have conversations with patients about immunisation. Currently, the Health Assessment (MBS item 701, 703, 705, 717) for people aged 75 years and older includes a review for influenza, tetanus, and pneumococcus vaccination history. While beyond the scope of the current study, it would be interesting to understand whether vaccination is on other care plans. As an example, the National Diabetes Services Scheme annual cycle of care checklist covers blood pressure, blood fats, foot assessments, eye examination, weight, and body mass, smoking etc. but does not mention immunisation. While this only represents one chronic disease, there may be others that could be targeted to ensure that the care plans include discussions about vaccination across the lifespan. Health workers need to be encouraged to discuss the benefits of vaccination in the same way that they promote the promotion of smoking cessation, programs to reduce blood pressure and cholesterol or mental health. As suggested by Doherty and colleagues, vaccination should be thought of as a health-promoting activity, rather than as a medical intervention targeted at one pathogen (20). Alternatively, it has been recommended that health practises could improve uptake of the recommend National Immunisation Program vaccines for patients with high risk conditions (e.g., diabetes, asthma, heart failure, and the elderly) by systematic monitoring the immunisation status of these patients (21).

The following are noted as limitations for this work: (1) interviews were only undertaken with a select group of participants, so the possibility of other important themes emerging cannot be ruled out; (2) the use of snowball recruitment may have also reduced the range of opinions amassed from participants; and (3) specific details regarding the participant's role was also not collected.

## Conclusion

While significant improvements have been made in adult immunisation uptake, there are still gaps across the program. While the system remains under stress because of the COVID-19 pandemic, it is not appropriate to implement any additional programs. Looking forward (and after reflecting on the lessons learnt from implementing and delivering the COVID-19 vaccination program), there is urgently a need to boost the use of surveillance data from the AIR to identify where gaps remain in adult vaccination, as well as advocate for changes to the AIR to facilitate the ability of providers to use AIR to identify adults at risk of being under-immunised. Beyond that there is a need to explore new options to promote activation of adults for vaccination via the use of patient care plans.

## Data Availability Statement

The raw data supporting the conclusions of this article will be made available by the authors, without undue reservation.

## Ethics Statement

The studies involving human participants were reviewed and approved by University of New South Wales HREAP G: Health, Medical, Community and Social. The participants provided verbal consent to participate in this study.

## Author Contributions

HS conceived the study, undertook the data analysis, interpretation, and with developing the paper. KM, AH, AD, and JK assisted with the development of the study, interpretation of results, and the final draft of the paper. All authors contributed to the article and approved the submitted version.

## Funding

This study received funding from Seqirus Australia. The funder was not involved in the study design, collection, analysis, interpretation of data, the writing of this article or the decision to submit it for publication.

## Conflict of Interest

HS has previously received funding from drug companies for investigator driven research and consulting fees to present at conferences/workshops and develop resources (Seqirus, GSK and Sanofi Pasteur). She has also participated in advisory board meeting for Sanofi Pasteur. AH has received honorarium from pharmaceutical companies for workshop presentations (GSK and MSD). The remaining authors declare that the research was conducted in the absence of any commercial or financial relationships that could be construed as a potential conflict of interest.

## Publisher's Note

All claims expressed in this article are solely those of the authors and do not necessarily represent those of their affiliated organizations, or those of the publisher, the editors and the reviewers. Any product that may be evaluated in this article, or claim that may be made by its manufacturer, is not guaranteed or endorsed by the publisher.
